# (Dis)confirming theories of consciousness and their predictions: towards a Lakatosian consciousness science

**DOI:** 10.1093/nc/niae012

**Published:** 2024-03-16

**Authors:** Niccolò Negro

**Affiliations:** School of Psychological Sciences, Tel Aviv University, Tel Aviv-Yafo 69978, Israel

**Keywords:** consciousness science, lakatos, confirmation theory, predictions, theory-appraisal

## Abstract

The neuroscience of consciousness is undergoing a significant empirical acceleration thanks to several adversarial collaborations that intend to test different predictions of rival theories of consciousness. In this context, it is important to pair consciousness science with confirmation theory, the philosophical discipline that explores the interaction between evidence and hypotheses, in order to understand how exactly, and to what extent, specific experiments are challenging or validating theories of consciousness. In this paper, I examine this intricate relationship by adopting a Lakatosian lens. I propose that Lakatos’ philosophy of science can aid consciousness scientists to better interpret adversarial collaborations in consciousness science and, more generally, to develop a confirmation-theoretic model of theory-appraisal in this field. I do so by suggesting that such a model be built upon three Lakatos-inspired criteria for assessing the relationship between empirical evidence and theoretical predictions: (i) the model should represent the ‘distinction between prediction and accommodation’; (ii) the model should represent the ‘structural relevance’ of predictions; (iii) the model should represent the ‘boldness’ of the predictions. I argue that a Lakatosian model of theory-appraisal has both normative and descriptive virtues, and can move the debate forward by acknowledging that theory-appraisal needs to consider the diachronic development of theories, their logical structure, and their relationship with background beliefs and knowledge.

## Introduction

This paper presents some philosophical insights on how to think about the (dis)confirmatory relationship between theories of consciousness and empirical evidence. Thinking seriously about the nature of this relationship is important because the neuroscience of consciousness is seeing a proliferation of theories (Del Pin et al. 2021, Signorelli et al. 2021, Seth and Bayne 2022), and consensus is far from near (Francken et al. 2022, Yaron et al. 2022).

Adversarial collaborations, which intend to test different theories of consciousness based on contrasting predictions (Melloni et al. 2021, 2023), promise to reduce the theory-space, and ultimately illuminate the details of how consciousness and brain activity relate. This is surely a welcomed empirical acceleration.

I argue that consciousness science would benefit from directly engaging with confirmation theory, the philosophical field studying the relationship between evidence and hypotheses (Hesse 1974, Godfrey-Smith 2003, Chalmers 2013, Crupi 2021), since conclusions about the epistemic solidity of theories of consciousness can be unwarranted if not paired with carefully constructed arguments that are sensitive to the nature of the relationship between evidence and theories.

Given that the urge of thinking seriously about confirmation theory in the context of consciousness science is motivated mainly by the goals and the results of adversarial collaborations, I will frame most of the discussion around the first experiment of this sort, designed and performed by the Cogitate Consortium (Cogitate et al. 2023), but my general intention is to provide some suggestions on how to navigate the broader debate on whether, and to what extent, empirical evidence can corroborate or disconfirm theories of consciousness. I will do so by building on Imre Lakatos’ view of scientific progress (Lakatos and Musgrave 1970, Lakatos 1976). I argue that some Lakatos-inspired criteria can help consciousness scientists build a confirmation-theoretic model of normative theory-appraisal for consciousness science. Despite this project being mainly philosophical, I believe it can be seen as complementary to formal approaches that intend to score through probability theory the degree of confirmation that empirical evidence provides to theories (Corcoran et al. 2023).

This project has both descriptive and normative components: it is primarily normative, insofar as it prescribes a way to interpret adversarial collaborations and to guide consciousness scientists towards specific philosophical problems that ought to be solved when building a model of theory-appraisal in consciousness science; and it is descriptive because the Lakatosian lens I apply to consciousness science is already implicit in what consciousness scientists do in practice. A comprehensive and systematic analysis of such practice is nevertheless missing, and that is the descriptive goal of this paper.

The aspiration of this paper is to start a conversation, not to settle it. It is possible that Lakatos’ philosophy of science is not the best way to interpret the evidential fit between theories of consciousness and experimental results, but I will build a case that a Lakatosian framework does seem to satisfactorily address the main concerns we should consider when trying to flesh out a confirmation-theoretic framework for consciousness science.

In the first section, I offer a potential reading of the philosophy of science behind theory testing in consciousness science, but I then argue that such a reading is problematic. In the second section, I suggest that we look at empirical theory testing through a Lakatosian lens, and I apply such a lens to consciousness science. In the third section, I specify three criteria that a Lakatos-inspired model of theory-appraisal for consciousness science should consider. In the Discussion, I evaluate the significance of this Lakatosian framework for empirical theory testing in consciousness science. A brief conclusion ends the paper.

## Instantia crucis and severe tests in consciousness science

In the *Novum Organum* (Bacon 1878), Francis Bacon introduced the notion of *instantia crucis* to refer to a situation able to determine the truth of a hypothesis and, at the same time, the falsity of alternative ones. Philosophers of science (Chalmers 2013, Crupi 2021) have seen in this approach the precursor of experimentalism (Hacking 1982, 1988, Mayo 1991, 1996), the view that the growth of scientific knowledge is driven by experiments, rather than theories, since scientists can design experiments to bring about a situation of this sort—the *experimentum crucis*.

In this view, scientific progress depends on eliminating predictions that do not conform well to evidence gathered through adequate and severe tests: the growth of scientific knowledge passes through an experimental situation that is able to ‘eliminate’ predictions derived from hypotheses under test. We can call this view ‘experimental eliminativism’.

An attractive idea (which I will later reject) is to think that this philosophy of science fits nicely with adversarial collaborations in consciousness science. An adversarial collaboration’s goal is to find convergence between proponents of different theories on how to design an experiment that could show a state of affairs that agrees with the prediction of one theory while disagreeing with the prediction of the rival (Clark et al. 2022). Thus, first we identify, with the help of proponents of rival theories of consciousness, an experiment in which leading theories of consciousness generate contrasting predictions; then, the experiment will decide which theory conforms better with the observed data (Melloni et al. 2023).

This seems to suggest that an assumption behind adversarial collaborations in consciousness science is that scientific progress passes through accumulation of experimental practice, in line with the main tenet of experimentalism in philosophy of science.

If we were to build a confirmation-theoretic model of theory-appraisal based on experimental eliminativism, adversarial collaborations would be seen as an ‘elimination race’: a theory is confirmed if it passes a severe test, and disconfirmed if it does not. Every experiment would correspond to a lap of the race, at the end of which one competitor is eliminated.

Despite some passages seem to suggest this view [e.g. Melloni and colleagues write that the adversarial collaboration between two theories ‘should yield reliable results that can provide substantial evidence for one or the other theory, in order to arbitrate between them’ (Melloni et al. 2023, p. 2)], I believe this interpretation would be inappropriate for both normative and descriptive reasons. Although in the literature adversarial collaborations have not been explicitly aligned with experimental eliminativism, this interpretation is sometimes implicit in how adversarial collaboration projects are presented in popular outlets (Finkel 2023, Lenharo 2023), and therefore my discussion aims at preventing such interpretation.

Let us focus first on the descriptive reasons for not aligning adversarial collaborations with experimental eliminativism. Take the specific collaboration testing different predictions from the Integrated Information Theory (IIT) (Tononi et al. 2016, Albantakis et al. 2023) and the Global Neuronal Workspace Theory (GNWT) (Dehaene and Changeux 2011, Mashour et al. 2020), led by the Cogitate Consortium. According to IIT, consciousness corresponds to the amount of irreducible causation that a physical system can potentially exert upon itself, which can be mathematically measured through a formalism called Φ. In contrast, for GNWT, consciousness is a property of a representation: a representation becomes conscious when it is broadcast into a ‘global workspace’ in which it becomes available to a host of consuming systems.

Members of the Cogitate Consortium have realized early on that adversaries do not concede easily the elimination of their theories, and rather try to adjust their view in light of the evidence (if they accept the evidence to begin with). This has led members of the Cogitate Consortium to align adversarial collaborations with a sophisticated form of falsificationism (Melloni 2022) by stating, e.g. that the purpose of their adversarial collaboration was ‘to falsify divergent predictions of IIT and GNWT and not to provide confirmatory evidence’ (Cogitate et al. 2023, p. 25), and that it was aimed at ‘providing the means to change one’s mind given contradictory results’ (Cogitate et al. 2023, p. 24).

Apart from these descriptive reasons, there are also normative reasons for not aligning adversarial collaborations in consciousness science with experimental eliminativism. This is because not every experiment in this context constitutes a ‘severe test’, in the eliminativist senses. To see why, we need to focus first on what a severe test is, and then on the specifics of the collaboration led by the Cogitate Consortium.

The most sophisticated version of experimental eliminativism has been arguably developed by Deborah Mayo (Mayo 1991). According to Mayo, a hypothesis passes a severe test if the (objective) probability of obtaining a specific experimental result is very high if that hypothesis is true and, crucially, very low if the hypothesis is false.

Imagine that we are interested in testing Snell’s law of refraction of light through refractive media. We can do so by measuring the angles of incidence and refraction and see whether they coincide with what Snell’s law predicts. Assume that they do, but our measurements come with quite large margins of error. In fact, it might happen that alternative theories of refraction (like Ptolemy’s theory) predict results that fall within those margins of error, and therefore could account for the experimental result. This means that the probability of obtaining that particular experimental result is quite high even if Snell’s law is false, because predictions of alternative theories are not eliminated by this specific measurement. This is why, according to Mayo’s view, the hypothesis (i.e. Snell’s law) is ‘not’ confirmed by this experiment: it does not pass a severe test.

Vice versa, the famous experiment led by Eddington and Dyson in 1919 (Dyson et al. 1920) for measuring the deflection of starlight in proximity to the sun corroborated Einstein’s theory of gravity because it was a severe test for that theory: Einstein’s prediction was that the light would deflect at the limb of the sun of 1.75 arc seconds, while the Newtonian theory predicted a deflection of 0.87 arc seconds. The two expeditions reported a deflection of 1.98 ± 0.18 arc seconds and 1.61 ± 0.45 arc seconds, so this was enough to eliminate the predictions of the Newtonian theory of gravity. In this case, then, we have a severe test because the probability of obtaining the results observed by Eddington would be quite low, had Einstein’s theory been false.

Do the experiments led by Cogitate have the same degree of ‘severity’? The Cogitate Consortium have tested these two theories based on three different predictions, but I will now focus mainly on the first. The first prediction is based on different brain areas involved in conscious perceptions: according to IIT, it should be possible to decode stimulus category and orientation, when that stimulus is consciously perceived, from posterior areas only (Boly et al. 2017), while for GNWT it should be necessary to include areas of the prefrontal cortex. Cogitate et al. (2023) have found that decoding of a consciously perceived stimulus is in fact maximal when based on posterior areas, as predicted by IIT.

Now, if we take experimental eliminativism (at least in Mayo’s sophisticated version) as the philosophy of science providing a confirmation-theoretic foundation for the science of consciousness, we would have to admit that this part of the first Cogitate experiment, by itself, is not quite a severe test. IIT and GNWT are not the only options available in the theoretical landscape of consciousness science, and several competing theories of consciousness also predict that frontal areas are necessary for conscious perception (Graziano and Webb 2015, Brown et al. 2019, Lau 2022), while some others predict that posterior areas are sufficient (Lamme 2006, 2010). This means that it is not true that there would be low probability of maximally decoding conscious contents, if GNWT were false (since one could find frontal decoding due to another mechanism that is not related to a global workspace—e.g. a higher order representation); and moreover, it is not the case that if IIT were false, then the probability of decoding conscious contents from posterior areas only would be low (since maximal decoding could depend on local recurrent activity). Therefore, Mayo’s severity criterion is not satisfied. But in this view, if an experiment is not a severe test, then it cannot contribute much to the growth of scientific knowledge.

My claim is that this conclusion, if applied to the series of experiments performed by the Cogitate Consortium, is unacceptable, because the experiments do challenge both theories, and push theorists to either dismiss the evidence for methodological limits (however, this option should be prevented by the very nature of adversarial collaborations) or adjust their theories in light of empirical evidence. Thus, the Cogitate experiment contributes to substantial progress in consciousness science despite not being a severe test in the experimental eliminativist sense.

There is a further problem with experimental eliminativism, which can be seen by analysing the other two predictions tested by the first Cogitate experiment: the second prediction was based on whether the neural activity that correlates with conscious contents is sustained for the duration of the experience (as predicted by IIT) or, instead, phasic, with spikes of activity correlating with stimulus onset and offset (as predicted by GNWT). The third prediction focused instead on interareal connectivity corresponding to conscious perception, with IIT predicting high interconnectivity between posterior regions for the duration of the experience, and GNWT predicting phasic connectivity between category selective areas and the prefrontal cortex.

The results here were mixed, with the second prediction favouring IIT, and the third prediction being more aligned with GNWT.

The crucial issue with experimental eliminativism is that it does not clearly specify how to connect these contrasting pieces of evidence with high-level theories. Here, I build on the idea that hypotheses can be formulated at various levels of analysis: they can be extremely specific and fine-grained, or they can be more general, less detailed, and function as working hypotheses. Or, they can be formulated at an even more general level as research programmes, theories, or paradigms (Douglas and Magnus 2013).

The alleged ‘elimination race’ posited by experimental eliminativism would be between low-level, very fine-grained, hypotheses (e.g. whether the hypothesis that neural activity during conscious perception is phasic should be rejected), but it is not clear whether, or at which point, the high-level theory generating that prediction (e.g. GNWT) should be rejected. Indeed, it is not clear whether ‘theories’ of consciousness could be part of the race at all. As Hacking (one of the founding figures of the experimentalist tradition) puts it, ‘experimentation has a life of its own’ (Hacking 1983, p. 150), largely independent of the theories that motivate it. This view is silent on how low-level experimental evidence connects to high-level theories [for criticisms of Mayo’s view along these lines, see Musgrave and Mayo (2009), Worrall and Mayo (2009), Douglas and Magnus (2013)]. Therefore, experimental eliminativism is unable to provide a picture of how scattered and sparse low-level evidence can inform our normative appraisal of theories of consciousness.

This discussion suggests that interpreting the philosophy of science at the basis of adversarial collaborations as an experimental eliminativism would be inappropriate, for both descriptive and normative reasons.

What we need is a philosophical view of scientific practice able to account for: (i) the informativeness of experiments carried out in the context of adversarial collaborations; and (ii) a unified picture of high-level theories and their low-level experimental predictions.

I believe that such a framework can be provided by applying the philosophy of science of Imre Lakatos to consciousness science. A Lakatos-inspired framework, I contend, could also help reframe our expectations from theory testing in consciousness science: we could perhaps overcome any ‘elimination’ talk (at least in the short term), and interpret instead experimental work as an opportunity for theoretical self-improvement [in the context of adversarial collaborations, this has been noticed by Cowan et al. (2020) and is very much in line with the sophisticated falsificationist attitude of Melloni (2022) and Cogitate et al. (2023)].

## Lakatos and the science of consciousness

In this section, I will briefly present the main ideas of Lakatos’ philosophy of science (Lakatos 1968a, 1968b, 1974, 1976, Lakatos and Musgrave 1970), and I will then reinterpret them in the context of consciousness science.

The Lakatosian view of science builds on the idea that scientific theories have two components: the ‘core’, namely the claims that characterize the essence of the theory, and the ‘belts of peripheral auxiliary hypotheses’. The conjunction of core theses and auxiliary hypotheses entails the theory’s predictions, and experimental testing targets these predictions.

Lakatos agreed with Popper (Popper 1959) that scientific progress is not made by verifying predictions entailed by theories, but by trying to falsify them. However, if a theory is made of a conjunction of core and auxiliaries, a falsified prediction does not automatically disconfirm the core claims of the theory, but rather it disconfirms the conjunction of core and auxiliaries, and a conjunction is false if only one of the conjuncts is false. This means that scientists can interpret the experimental falsification of a prediction by holding on to the core claims of their theory and by rejecting some of the auxiliaries. This makes theory testing an arduous affair, since, as Duhem and Quine realised, it becomes difficult to disentangle the theoretical aspects put under pressure by empirical testing from all the background beliefs held by scientists (Quine 1951, Duhem 1954).

Lakatos’ view, which was substantially informed by the history of science, is that scientists generally opt for revising some of the auxiliary hypotheses while holding on to the core claims of the theory. This generates a ‘research programme’, a diachronically constituted scientific effort built around a core set of theses, assumptions, and heuristics: within a research programme, the core remains the same, while the belts of peripheral auxiliary hypotheses are modified under the pressure of empirical testing.

Now, scientists can modify the theory by changing its empirical or theoretical content. The ‘theoretical’ content of the research programme requires that the new theory generates novel testable predictions, while the ‘empirical’ content requires that at least some of these novel predictions be true. If a research programme fails in at least one of these two aspects, it can be said to be ‘degenerating’, rather than ‘progressive’, and therefore it does not constitute good science [for an historical case, see Scerri and Worrall (2001)]. However, for Lakatos, a degenerating research programme can ultimately be discarded only by ‘another research programme’, not by an experiment (Lakatos 1974).

This brief presentation of Lakatos’ view should suffice to see why a Lakatosian lens on consciousness science can solve the problems that emerged by interpreting theory testing through the experimental eliminativist lens.

First, experiments like the one designed and performed by the Cogitate Consortium are informative and contribute substantially to the growth of knowledge in consciousness science, insofar as they provide a falsification-driven test for the empirical content of consciousness research programmes and constrain the possible moves theorists can make to adjust their theories. This is actually hinted at by the members of the Consortium, who write that the adversarial collaboration was aimed at ‘providing the means to change one’s mind given contradictory results’ (Cogitate et al. 2023, p. 24). Experiments are thus seen as informative as long as they have the potential to disrupt the empirical content of the theory and therefore generate a research programme that, in the longer run, can turn out to be progressive or degenerating. The Lakatosian framework seems to capture quite well the intent of consciousness scientists actively involved in this adversarial collaboration, which is a positive descriptive virtue of the framework.

Second, the Lakatosian framework admits that scientists can in fact be rational when they hold on to their theory even when presented with some contrasting evidence. Again, this is because a refuted prediction can simply signal that the specific connection that the scientist drew between core claims and auxiliary hypotheses is incorrect; it does not necessarily mean that the theoretical core is wrong. This is descriptively accurate too: e.g. in the work by Cogitate et al. (2023, p. 27), Dehaene defends GNWT in light of some contrasting evidence, and claims that a proper evaluation of the theories should wait for the second Cogitate experiment (Melloni et al. 2023). And normatively speaking, if the Lakatosian framework is on the right track, he is entirely rational in doing so—see also Fazekas et al. (2024).

Third, the connection between theory and prediction is a natural consequence of the distinction between core and protective belts of auxiliaries (or between centre and periphery): predictions are (deductively) derived from the conjunction of core and auxiliary hypotheses, which means that, as Musgrave (2023) notes, auxiliary hypotheses have two jobs in the Lakatosian picture: on the one hand, they protect the core claims from ‘direct’ falsification; on the other hand, they connect theoretical claims with experimental practice. A theory will eventually be refuted when its core will be unable to parsimoniously relate to empirical evidence and background knowledge, and will be superseded in that regard by a rival theory: the normative appraisal of research programmes depends on how well low-level predictions do experimentally, ‘and’ on how well the scientists will be able to modify the theory in light of experimental evidence.

The Lakatosian framework is thus quite promising in interpreting adversarial collaborations in consciousness science and can shed light on the general nature between evidence and hypotheses. I believe that this framework is conducive to the claim that adversarial collaborations in consciousness science should be seen as theoretical self-improvement rather than as an ‘exclusion race’. This, in turn, can inform a more general confirmation-theoretic view on how experimental evidence corroborates high-level theories of consciousness.

In order to use this framework to build a model of theory-appraisal for consciousness science it is crucial to specify the Lakatos-inspired criteria upon which this model could/should be built. These criteria are: (i) the model should represent the ‘distinction between prediction and accommodation’; (ii) the model should represent the ‘structural relevance’ of predictions; (iii) the model should represent the ‘boldness’ of the predictions.

## Three Lakatosian criteria for theory-appraisal

In this section, I briefly introduce the criteria, inspired by the Lakatosian view of science, that can be used to build a model of theory-appraisal for the neuroscience of consciousness. These criteria are probably not exhaustive and are sketched here at a general level so as to point at the type of philosophical considerations that could be beneficial to consciousness scientists interested in building a model of theory-appraisal. The specific approach one takes with respect to these criteria is then a modeller’s decision, and it is possible that different formal models can be built by following the same set of criteria. My goal here is to just flag that whatever choice the modeller makes, it will probably come with substantial philosophical assumptions, and so I will make clear what these assumptions amount to.

It is important to clarify that these confirmation-theoretic criteria are not all explicitly stated by Lakatos but are rather extrapolated from his overall view. The resulting model is, then, not a strict application of Lakatos’ view to consciousness science, but a more liberal reinterpretation that, despite being generally inspired by his account, could be complemented by theoretical elements of non-Lakatosian accounts of scientific progress.

### Prediction vs accommodation

Philosophers of science tend to distinguish predictions from accommodations. To use Nozick’s vivid example, imagine two archers: one hits the bullseye, while the other hits a random white wall and then draws a bullseye around the arrow (Nozick 1983, p. 9). The first would be like a predictor, the second like an accommodator.

Predictivism is the philosophical view that predictions bear higher confirmatory power than accommodations (Maher 1988, Worrall 1989, Lipton 1990, Douglas 2009, Douglas and Magnus 2013), and it seems to be at the core of adversarial collaborations in consciousness science because theories are not supposed to be tested through pieces of evidence that they can accommodate, but by evidence they can predict.

The distinction is particularly relevant for a Lakatosian framework: as seen in the previous section, progressive research programmes are able to predict novel facts, while degenerating programmes fail to do so. The notion of ‘novel’ fact is thus crucial to evaluate the progressivity of a research programme, and since progressivity is based on predictions, rather than accommodations, the distinction between prediction and accommodation rests on what ‘novel’ means.

At first glance, it might not seem troubling: a prediction is about a state of affairs the occurrence of which is unknown at the time of the formulation of the prediction, while an accommodation occurs when the state of affairs is already known at the time a theory is formulated, and the theory is able to account for it. This is the ‘temporal’ reading of the prediction/accommodation distinction, based on the view that novel facts are temporally novel, and is arguably what Popper (1968) and Lakatos (1968b) had in mind.

However, the case of Mercury’s perihelion, an anomaly in the Newtonian theory of gravity, poses a problem for this temporal view of ‘novel facts’. The anomaly of Mercury’s perihelion was well known ‘before’ Einstein’s theory of gravity came along; and yet, Einstein included the measurement of the precession of Mercury’s perihelion as one of the three key tests for general relativity, because if the theory was right, the curvature of spacetime would have naturally accounted for the precession (Einstein 1916). Einstein’s view was that it would be wrong to think that the precession of Mercury’s perihelion does not have much confirmatory power with respect to general relativity: the anomalous behaviour of Mercury’s perihelion just naturally follows from general relativity, and therefore it confirms the theory even if it was already known when the theory was formulated.

Lakatos, following Zahar (Zahar 1973, Lakatos and Zahar 1975), accepted Einstein’s suggestion that novelty is not a temporal notion, and modified his theory by claiming that a fact is novel insofar as it was not part of the empirical facts that motivated the construction of the theory in the first place. Einstein did not build general relativity to explain Mercury’s perihelion, and that is why general relativity does not accommodate the precession of Mercury’s perihelion. Rather, it (successfully) predicts it. This is what philosophers of science call ‘use-novelty’ or ‘heuristic-novelty’—for a similar view, see Worrall (1985, 1989).

This debate can be translated into consciousness science. Consider the results of Sperling’s partial report paradigm (Sperling 1960), where Sperling demonstrated that, even if subjects can normally report four or five items out of a larger array of letters, organised in a 3 by 3 or 4 by 4 matrix, they are nonetheless able to report almost all the letters in a row if a row is cued immediately after the array of letters disappears. This happens independently of the cued row, suggesting that subjects are conscious of more than what they can normally report (Block 2011)—for an alternative interpretation, see Phillips (2011).

IIT was not built to account for the results obtained in Sperling’s partial report experiments, but such results can be accounted for by IIT quite naturally (Haun et al. 2017, Tononi 2015, Tononi et al. 2016, pp. 456–457). In fact, Tononi (2015) writes that

IIT emphasizes that the set of elements (neurons) that specify a particular concept within a conceptual structure may be more or less difficult to access from within the complex […]. The concepts that can be accessed and communicated to an external observer at any given time are a minimal subset of the entire set of concepts that compose the quale sensu lato […] nor can one easily communicate the relationships among them (distance in cause-effect space) that give each experience its particular meaning. (In the current version of the theory (Albantakis et al. 2023), ‘concepts’ are called ‘phenomenal distinctions’ and conceptual structures are ‘cause-effect structures’, or ‘Φ-structures’.)

This suggests that IIT predicts that phenomenal experience (i.e. the overall cause-effect structure composed of sub-structures and relations among them) goes beyond what can be reported (Block 2011), and can thus account for Sperling’s results.

So, Sperling’s experimental results count as a ‘use-novel’ fact for IIT, even if the Sperling experiment predates the development of IIT by about four decades. But take now Lamme’s recurrent processing theory (RPT) (Lamme 2010), the view that feedback loops within sensory areas are necessary and sufficient for sensory consciousness. Lamme (2006, 2010) states that Sperling’s findings are among the motivating grounds for his theory, and this means that Sperling’s experimental results are accommodated, and not predicted, by RPT.

The opposite is true for the evidence that the cerebellum can be removed without impacting consciousness. Even if the foundation of IIT is phenomenological (Oizumi et al. 2014, Ellia et al. 2021), the fact that the cerebellum is not necessary for consciousness is often presented by IIT proponents as one of the facts that a theory of consciousness needs to explain, and is therefore part of the empirical motivating ground for the development of IIT (Massimini and Tononi 2018). If this is right, then the fact that the cerebellum is not necessary for consciousness is accommodated by IIT, but it counts as a ‘use-novel’ fact for RPT, because RPT can easily explain this fact by pointing out that the neuroanatomical connectivity of the cerebellum is mostly based on feedforward connections. In this case, then, the fact that the cerebellum is not necessary for consciousness is confirmatory for RPT, but not (or only partially, if accommodations have some confirmatory value) for IIT.

The present discussion indicates that a general model of theory-appraisal should be sensitive not only to the logical relation between evidence and hypotheses, but also to how, and in which context, the hypothesis is formulated. The reason why this discussion is relevant is that if an experiment (either based on adversarial collaboration or not) tests only some theories of consciousness at a time, it might be possible that a correct prediction of a theory ‘T’ could be easily accounted for by an alternative theory ‘T1’ that is not directly tested in that specific experiment. That would count as a novel correct prediction (both in temporal and ‘use-novelty’ sense) for T, but also as a use-novel correct prediction for T1: experiments could have confirmatory value for theories that are not directly tested in that particular setting.

There is a further complication, though. Consider Mashour et al.’s claim (Mashour et al. 2020, p. 787) that GNWT can easily explain the finding, supposedly supporting IIT, that the reduction in complexity of brain activity can reliably predict whether an unresponsive subject is dreaming, in a state of unresponsive wakefulness, or non-conscious (Massimini et al. 2009, Casali et al. 2013). Mashour et al. write that ‘Although this experiment was inspired by an alternative theory of consciousness, […] the results are fully compatible with the GNW, which predicts that the conscious state leads to a deeper and more prolonged propagation of activation through long-distance connections compared to the unconscious state’ (Mashour et al. 2020, p. 787). Although there might be issues with how to formally interpret the notion of brain complexity, and therefore on whether there is convergence between the formalism that is supposed to support IIT and its compatibility with GNWT (Sarasso et al. 2021, Farisco and Changeux 2023), we can accept for the sake of the argument that these experimental findings do count as a use-novel fact for GNWT. If that is the case, they should be confirmatory relevant for GNWT under a ‘use-novel’ framework.

However, a Lakatosian scholar like Nunan (1984) has argued that a fact can count as novel only if it is not predicted by an alternative research programme, on the basis that such a novelty criterion is the only one that is able to connect the progressivity of a research programme with rival programmes, and therefore able to demonstrate why the progressive research programme should be rationally chosen over the rivals (see also Lakatos 1974). In a similar spirit, Douglas and Magnus (2013) write that ‘new evidence or new evidential relations throw an evidential gauntlet down for competitor theories — they must *accommodate* the new evidence or risk epistemic demerit’ (Douglas and Magnus 2013, p. 13; emphasis added). If this is right, GNWT would ‘not’ predict the experimental finding of Casali et al. (2013), not even in a ‘use-novel’ sense—it would merely accommodate it, and therefore that empirical evidence could not contribute much to confirming GNWT.

Here, my goal is not to defend a specific way of drawing the distinction between prediction and accommodation, but to flag some crucial questions that are preliminary to any model of theory-appraisal that can be employed in evaluating and comparing theories of consciousness. It is important that a model of theory-appraisal be sensitive to these concerns, and it will ultimately be a theoretical (and philosophical) choice of the modeller how to specifically interpret the novelty of a prediction, and how to value the confirmatory aspect of accommodations.

### The structural relevance of predictions

The second criterion builds upon the Lakatosian distinction between the core theses of a theory and its auxiliary hypotheses. This is relevant to theory testing in consciousness science, because some have remarked that the first adversarial collaboration testing contrasting predictions from IIT and GNWT did not test the theories directly. For example, Dehaene writes that ‘none of the massive mathematical backbone of IIT, such as the φ measure of awareness, was tested in the present experiment’ (Cogitate et al. 2023, p. 27)—see also Fleming (2023). However, a similar point could be raised for GNWT too, as correctly pointed out by Seth (2023) and Fazekas et al. (2024).

If we take the Lakatosian picture seriously, this worry is not a concern *per se*, but just the rule of theory testing: scientific theories are not tested at their core, but rather through the belts (which are called ‘protective’ precisely for this reason) of auxiliary hypotheses.

This is also the lesson learnt from the Duhem-Quine thesis: given the immense web of beliefs held by scientists, it is always possible to fit the data with any theory by adjusting and modifying the related auxiliaries, and therefore a ‘direct’ empirical test of theoretical claims is fundamentally impossible.

My claim here is that we can circumvent this concern by noticing that ‘the degree of confirmation a prediction confers to a theory partly depends on how peripheral the prediction is’: the farther away from the core the prediction is, the less (dis)confirmatory power will bear.

To substantiate this discussion, let us represent (at a general level) the structure of IIT and GNWT through a Lakatosian lens. The basic idea is that each level of the periphery generates predictions based on what is assumed and predicted by the previous level, with more peripheral levels specifying at a finer grain the predictions of the levels closer to the centre, thus making the theory more detailed—for similar views, see Henderson et al. (2010) and Grim et al. (2022).

For IIT, the core claims are its phenomenological axioms (which define the essential properties of consciousness), its postulates (which state how those properties can be accounted for in physical terms), and the explanatory identity stating that consciousness is integrated information (Albantakis et al. 2023). Outside of the core we can find the mathematical translation of the postulates, and the claim that consciousness can be measured through the ‘specific’ formal apparatus of the theory. Notice that the ‘general level’ claim that consciousness can be quantitatively measured through integrated information is at the core of IIT, but the specific claim that consciousness is identical to the specific formalism presented by Albantakis et al. (2023) is outside of the core: the specific formalism to determine Φ has changed throughout the various iterations of the theory (Tononi 2004, 2012, Oizumi et al. 2014, Barbosa et al. 2021), while the general idea that consciousness is integrated information has remained constant.

In IIT, then, the first belt of auxiliaries just requires background knowledge of mathematical nature to derive the specific Φ-measure from IIT’s core.

From this first belt of auxiliary hypotheses, we can move to the next, and the distance we travel from the core will depend mainly on the number of auxiliary background assumptions we need to add to core claims in order to derive a specific prediction. For example, IIT’s prediction that the back of the brain should be sufficient for conscious perception depends on ‘neuroanatomical’ background knowledge telling us that posterior cortical areas, and not the prefrontal cortex, are constituted by neuronal grids that have the appropriate physical structure to sustain large Φ values (Grasso et al. 2021). Thus, to derive the prediction that the back of the brain is sufficient for conscious perception we need the specific mathematical formalism specified by the first peripheral belt ‘and’ some neuroanatomical background knowledge. In this way, we move from the first belt of auxiliary hypotheses to the second belt.

A similar analysis can be done with respect to GNWT: its core claim is of cognitive nature and states that conscious perception occurs when information processed by modular and local processors is broadcast into a global workspace which renders it available to various consumer systems (Baars 1988). This core claim is then made more precise by postulating that the neuronal implementation of the global workspace requires pyramidal neurons with long-range axons (Dehaene et al. 1998), which requires auxiliaries of neurophysiological nature. The theory can then predict that the prefrontal cortex is necessary for consciousness because it exhibits ‘the greater density of neurons thought to be critical for global broadcasting of information’ (Mashour et al. 2020, p. 777), and this prediction requires neuroanatomical auxiliaries [for in-depth discussions of the structure and historical development of the global workspace hypothesis, see Mashour et al. (2020, p. 776) and Baars et al. (2021)].

This discussion matters for adversarial collaborations because it is possible that a successful prediction might corroborate one theory while disconfirming the other, but the extent to which it does so might depend on how close that prediction is to the core of each theory, and possibly on the nature of the prediction, given the nature of the core [e.g. computational background assumptions might be more relevant than assumptions on the mechanistic details of the brain, if the core of the theory is computational—see Lau and Rosenthal (2011), Fleming (2020), Wiese and Friston (2021), Lau (2022)]: the idea is that the relevance of a prediction is associated to the disruptive power the prediction bears, and the closer the prediction is to the core, the higher the power (compare: the Mayor of London is probably aware that closing Liverpool Street Station causes much more troubles to their city than closing Cockfosters Station).

Imagine an experiment involving a prediction that makes use of certain assumptions on the link between attention and consciousness. The result of that experiment will be much more relevant for theories that see consciousness and attention closely related (Parr et al. 2019, Graziano 2022, Vilas et al. 2022), but only partially relevant for theories like IIT, which are not built around a connection between consciousness and cognition, and therefore require a large number of background assumptions to formulate predictions about the relation between consciousness and specific cognitive phenomena like attention. In case of contradictory evidence, IIT would just need to revise some auxiliaries about the relationship between integrated information and attentional processes, which would not significantly impact its core claims, and for this reason, although the experiment would still be informative, the disruptive power of the prediction would be limited.

The criterion of structural relevance thus maintains that a model of theory-appraisal should consider the distance between core claims and the peripheral level at which the experimental prediction is formulated: the longer the distance, the less relevant the prediction in confirming or disconfirming a theory.

### The boldness of predictions

The third Lakatosian criterion for a model of theory-appraisal stems from the Popperian tenet that good scientific theories should exhibit an element of risk: the criterion states that the confirmatory power of a prediction is partly constituted by its ‘boldness’, where boldness is intended as a measure of divergence from consensus and background knowledge. A bold prediction is a prediction that is peculiar to one theory, that sets it apart from competitors, and it would be very unlikely to be verified if the theory were false. (There is evidence from psychological research that people value bold hypotheses more than relatively uninformative ones (McKenzie and Amin 2002). This psychological fact can be nicely captured by the Lakatosian model I am suggesting, which speaks in favour of the model.) Predictions are bold when they are risky (i.e. depart from consensus) and meaningful (i.e. they single out a specific theory). (Special thanks to Liad Mudrik for pointing out this distinction to me.) As Lipton puts it, ‘evidence that discriminates between competing theories is more valuable than evidence that is compatible with all of them’ (Lipton 1990, p. 54).

There is a sense in which this criterion aligns with Mayo’s intuition that the confirmatory status of a prediction depends on a test that minimizes the chance that the prediction could turn out to be correct even if the theory is false: we want a prediction to be confirmatory of a specific theory because it is entailed by that theory and ‘that theory alone’. (Although there is conceptual alignment between the boldness of a prediction and Mayo’s notion of severity, in her view, the severity of the test is not complementary to the novelty of the prediction. Rather, her account intends to demonstrate that a prediction is confirmatory relevant only when it passes a severe test, and novelty is neither necessary nor sufficient for confirmatory power. Moreover, severe tests can target quite peripheral predictions.)

For this reason, boldness goes hand in hand with the idea of ‘precision’, although the two concepts are not exactly equivalent. The idea is that a prediction is precise when it limits vagueness, and it is clear in discriminating between prohibited states of affairs—the larger and better defined the set of prohibitions, the more precise the prediction is. For example, predicting that tomorrow will be sunny and windy is more precise than predicting that tomorrow will be sunny, but it might not be a bold prediction, if several different meteorological models predict that tomorrow will be sunny and windy. Thus, boldness discriminates between ‘theories’, given the predicted evidence, while precision discriminates between ‘states of affairs’. Despite this difference, the two concepts are related because it is easier for an imprecise prediction to be accounted for by a larger set of theories, since imprecise predictions rule out fewer states of affairs than precise ones [for the idea that imprecise predictions have epistemic value nonetheless, see Elliott-Graves (2020)]. Here, I will formulate the boldness criterion assuming that bold predictions must also be precise predictions.

To see how the criterion works in practice, consider another prediction of IIT. This is the quite surprising prediction that a network of inactive (but not deactivated) neurons can contribute to consciousness [for a critical discussion that questions whether this prediction is testable at all, see Bartlett (2022)]. The prediction derives from core claims of IIT, like the idea that what matters for consciousness is the intrinsic causal ‘powers’ (i.e. how neurons ‘can’ influence other neurons, rather than ‘whether’ they do so) of a physical system, together with auxiliary assumptions that bridge the general-level claims in the theory’s core to the neurophysiological context, and specifically the brain’s metabolic constraints (Balduzzi and Tononi 2009, p. 15).

This is a bold prediction of IIT, in the sense that the idea that inactive neurons can sustain consciousness as much as active ones is a ‘unicum’ among neuroscientific theories of consciousness, and therefore sets IIT apart from neuroscientific consensus. Tononi and Koch are aware of this, and write that ‘A theory is the more powerful the more it makes correct predictions that violate prior expectations’ (Tononi and Koch 2015, p. 9).

A final point on the relation between boldness and structural relevance: particular predictions that set apart a theory in the theory-space, and therefore score high in boldness, should be expected to be quite close to the core, and therefore score high in structural relevance. This is because the structural relevance of a prediction partly depends on the number of auxiliary background assumptions used to derive that prediction, and it is easier to formulate a hypothesis that substantially diverges from consensus and background knowledge if its construction is influenced more by the ‘gravitational’ pull of core claims, rather than by large part of more commonly shared background knowledge.

However, the two concepts remain distinct, since structural relevance refers to the informativeness of a prediction with respect to the theory’s core, while boldness refers to the informativeness of a prediction with respect to other theories in the theory-space. And given this independence, it might be possible that certain predictions that are quite peripheric turn out to be bolder than certain predictions that are closer to the centre.

## Discussion

### Applying the Lakatosian model to the cogitate experiment

How can these three criteria be practically applied to theory-appraisal in consciousness science? Although it is premature to perform a comprehensive assessment, it is possible to analyse how the Lakatos model presented above relates to the first Cogitate experiment. Again, the experiment tested three different and contrasting predictions of IIT and GNWT: the first tested the necessity of prefrontal activation for conscious perception (posited by GNWT and denied by IIT), the second examined whether the temporal activity associated with conscious perception is sustained (as predicted by IIT) or phasic (as predicted by GNWT), and the third tested whether the interareal connectivity during conscious perception was limited to sensory and category-specific areas in the posterior cortex (as predicted by IIT) or whether it extended to the prefrontal cortex (as predicted by GNWT).

The (dis)confirmatory relevance of these predictions can be assessed through the Lakatos-inspired criteria proposed here. First, all these predictions seem to be novel, both in the temporal and in the use-novel sense. In fact, the predictions are about states of affairs that are not known at the time of their formulation, and do not belong to the motivating grounds of IIT and GNWT. Second, all three predictions are quite peripheral, insofar as they are formulated at the neural level, and therefore require a considerable number of ‘implementation-specific’ auxiliaries. This is because the cores that generate them are formulated either at the functional (for GNWT) or at the phenomenological (for IIT) level. Third, the boldness of these three predictions seems to be limited. Although they do exhibit some elements of ‘risk’ (i.e. they clearly prohibit precise states of affairs), the predictions tested in the first Cogitate experiment could also be predicted by other theories of consciousness, and therefore they are not particularly ‘meaningful’ for the theories tested. As seen in Section 1, frameworks other than GNWT posit the necessity of the prefrontal cortex for conscious perception, and frameworks other than IIT deny it. This means that alternative frameworks could formulate predictions that overlap with the first and third prediction tested by the Cogitate experiment. Moreover, different accounts of time-consciousness (Kent and Wittmann 2021, Singhal and Srinivasan 2021) could potentially agree with either IIT or GNWT on the specific formulation of the second prediction, without necessarily agreeing with the whole theory. This suggests that the evidence does not strongly discriminate between different theories of consciousness and could be compatible with many of them: the boldness level of the predictions tested by the Cogitate experiment seems to be quite low.

Given the peripheral nature of the predictions tested, and their low boldness score, the Lakatosian model concludes that the impact of the Cogitate experiment on the whole theoretical architecture of IIT and GNWT is limited. Despite this limitation, the model posits that these types of experiments are necessary to drive the diachronic development of research programmes. Indeed, the model predicts that proponents of IIT and GNWT will quickly account for the contradictory evidence by revising auxiliary assumptions while leaving the core intact, and an assessment of these programmes will depend on whether the reactions of IIT and GNWT proponents will add to the empirical content of their theories or not.

For example, Dehaene writes that challenging results ‘do not seem unsurmountable given experimental limitations’ and questions whether subjects were indeed conscious of the stimuli whose orientation was not decoded from the prefrontal cortex (Cogitate et al. 2023, p. 27). On the other hand, IIT proponents defend IIT’s third prediction (challenged by the Cogitate experiment’s result) by pointing out that too few electrodes were used to record the signal from posterior areas. Thus, contradictory evidence is accounted for by revising auxiliaries related to experimental design—for discussion, see Fazekas et al. (2024, p. 6). These reactions do lead to more testable empirical content, since testable predictions can be derived from these methodological adjustments of IIT and GNWT proponents, and therefore the Lakatosian model concludes that, although a comprehensive assessment should be made after an iterative process of testing and revisions, both IIT and GNWT remain, at this stage, progressive research programmes.

### Lakatos and Bayes

My discussion here has been qualitative, as I focused on the nature of the confirmatory relationship between evidence and theories, but the discussion has been built on the idea that there can be ‘degrees’ of (dis)confirmation. The idea that a prediction comes with an associated value of confirmatory power, and that such value can be mathematically formalised, is generally the hallmark of Bayesian confirmation theory (Howson and Urbach 1989). It is thus reasonable to think that a formal confirmation-theoretic model of theory-appraisal will be based on some form of Bayesianism and will prescribe that consciousness scientists are rational as long as they modify their prior beliefs according to Bayes’ conditionalization rule (Corcoran et al. 2023).

A further question concerns the specific way that the concepts introduced here (e.g. disruptive power, boldness, etc.) can be precisely quantified, which is a topic that could be explored in future work.

As I said above, these Lakatos-inspired criteria do not intend to resolve the discussion about how to model the relationship between evidence and hypotheses in consciousness science, and it might very well be that a Bayesian account could complement and ameliorate the Lakatosian framework presented here [even if a full-blown combination of Lakatos and Bayes might be hard to achieve, see Lakatos (1968a)]. However, I stress that the importance of building a confirmation-theoretic model of theory-appraisal in consciousness science upon Lakatosian guidelines lies in the emphasis that this framework puts on the structural nature of theories, and on the idea that the normative appraisal of theories does not only depend on the relationship between evidence and hypotheses, but also on the diachronic development of the research programme.

### Elimination, subjectivity, and the relativism of theory-appraisal

It might be argued that substituting the emphasis on the ‘elimination’ of theories in favour of an assessment based on their progressivity risks losing an objective way to determine when it is rational to abandon a research programme. Critics of Lakatos have indeed pointed out that his demarcation criterion seems to allow scientists to retain a theory that keeps being disproven (Chalmers 2013, Musgrave 2023). Moreover, the Lakatosian account does not seem to provide a clear criterion to adjudicate between two competing programmes, if they are both progressive (Nunan 1984). Both these points suggest that the Lakatosian demarcation criterion for theory-appraisal might not be as objective as Lakatos wanted it to be, and that instead a certain amount of relativism and subjective judgement must play a role in theory-appraisal (Musgrave 1976, Laudan 1977, Feyerabend 1988).

The Lakatos-inspired model I am proposing might inherit the same weaknesses. However, the model proposed here can be complemented by non-Lakatosian elements, under the assumption that they cohere well with the Lakatosian view. For example, we can distinguish between the ‘criterion’ used for theory-appraisal and the ‘judgment’ on whether a research programme must be abandoned. I argue that the Lakatosian criterion remains objective, since what discriminates between progressive and degenerating research programmes is whether the choices scientists make in face of contradictory evidence lead to new empirical content (i.e. testable predictions) or not. On the other hand, the judgment on whether and when a programme should be abandoned will likely be influenced by sociological and pragmatic factors. These factors, despite generating some relativism and vagueness in the demarcation judgment in virtue of their non-empirical nature, are not entirely subjective, but intersubjective choices driven by an objective criterion for theory-appraisal. That is, sociological and pragmatic considerations might indeed influence the decision of consciousness researchers on whether to abandon or retain a research programme, but only at a second stage, when the process of empirical testing and revision has already established, through an objective criterion, which programmes are progressing, and which are degenerating.

Although this Lakatosian model helps avoid any ‘elimination’ talk in the short term, it is still able to provide guidance on how research programmes should be abandoned in the long term. In this sense, the model does not diminish the need for empirical stringency, but admits that scientific progress can be obtained at different stages, with both empirical (i.e. objective) and non-empirical factors playing a role at different stages. Admitting that sociological and pragmatic factors play a role in the demarcation judgment, then, does not detract from the utility of the Lakatos-inspired model proposed here.

Moreover, this lens opens the door to an interpretation of scientific progress based on the social and historical situatedness of scientists. As rightly noted by Michela Massimi, scientific agreement is ‘a fundamentally social and cooperative inquiry, where progress takes place not *in spite of* but *thanks to* a plurality of scientific perspectives’. (Massimi 2021, p. S6124; italics in the original).

This is very much in line with how theories of consciousness are tested, especially thanks to adversarial collaborations. In fact, some of the Lakatosian ideas I am suggesting have been more or less explicitly endorsed by scientists involved in adversarial collaborations. This speaks in favour of the picture I am proposing, which can be seen as a way to systematize and ground on philosophical foundations what is already done (at least in part) by consciousness scientists.

In sum, the Lakatosian picture sketched here can (i) provide a philosophical foundation to interpret the effort of adversarial collaborations in consciousness science by acknowledging the informativeness and relevance of experiments carried out in the context of adversarial collaborations; (ii) provide normative guidance on how to better interpret the nature of the relationship between evidence and hypotheses, and thus support the development of a model of theory-appraisal; (iii) explain why consciousness scientists are not dismissing their theories in light of some contradictory evidence, and prescribe that they are rational in doing so; and (iv) accurately describe the intentions and the practices of scientists who are actively testing theories of consciousness.

## Conclusion

In this paper, I have provided a philosophical foundation for the debate of how theories of consciousness fit with empirical evidence. This has been motivated by the objective of setting the stage for the development of a confirmation-theoretic model of theory-appraisal for consciousness science.

I have argued that the Lakatosian framework I am suggesting can help the development of a model of theory-appraisal by considering three different criteria of how empirical evidence relates to theories: these are (i) the distinction between prediction and accommodation; (ii) the structural relevance of predictions; and (iii) the boldness of predictions.

The picture depicted here sees empirical theory testing as an opportunity for theoretical self-improvement, where scientific progress is ultimately driven by the ability of scientists to collaboratively devise ways to look for contradictory evidence and ameliorate their theories in light of such evidence.

A discussion on the nature of the relationship between evidence and theories is particularly important in a field like consciousness science, where theories have been traditionally built and tested in theoretical silos, and adversarial collaborations are challenging theorists to revise their theories given the evidence. Such an empirical acceleration is surely beneficial, and the present paper intends to sketch a possible way to philosophically complement this empirically driven development of the neuroscience of consciousness and its theories.

## Data Availability

No new data were generated or analysed in support of this research.
